# Management and Outcome of Invasive Clindamycin-Resistant MRSA Community-Associated Infections in Children

**DOI:** 10.3390/antibiotics14010107

**Published:** 2025-01-20

**Authors:** Amanda E. Macias, Grant Stimes, Sheldon L. Kaplan, Jesus G. Vallejo, Kristina G. Hulten, J. Chase McNeil

**Affiliations:** 1 Division of Infectious Diseases, Department of Pediatrics, Baylor College of Medicine, Houston, TX 77030, USA; amanda.macias@bcm.edu (A.E.M.); grant.stimes@bcm.edu (G.S.); skaplan@bcm.edu (S.L.K.); jvallejo@bcm.edu (J.G.V.); khulten@bcm.edu (K.G.H.); 2 Pediatric Infectious Diseases, Texas Children’s Hospital, Houston, TX 77030, USA; 3 Department of Pharmacy, Texas Children’s Hospital, Houston, TX 77030, USA

**Keywords:** MRSA, invasive, clindamycin resistant, children

## Abstract

Background: Clindamycin resistance among community-associated methicillin-resistant *Staphylococcus aureus* (CA-MRSA) complicates the management of a challenging infection. Little data exist to guide clinicians in the management of invasive clindamycin-resistant CA-MRSA infections in children and studies using oral regimens such as trimethoprim-sulfamethoxazole (TMP-SMX) and linezolid for treatment of these infections are limited. We sought to reevaluate antibiotic management among invasive CA-MRSA at a tertiary children’s hospital. Methods: Cases of invasive clindamycin-resistant MRSA infections in children were identified through an ongoing *S. aureus* surveillance study. Eligible cases were those occurring in otherwise healthy children from 2011–2021. Medical records were reviewed. Results: Thirty-four subjects met inclusion criteria. The most common diagnoses were osteomyelitis (n = 17) and deep abscess (n = 7). The median duration of IV therapy was 11.5 days (IQR 6–42 days) and total therapy (IV + oral) was 32 days (IQR 23–42). Overall, 50% of patients were transitioned to oral therapy. Definitive antibiotics used for treatment included vancomycin (n = 15), TMP/SMX (n = 9), linezolid (n = 7), ceftaroline (n = 2), and doxycycline (n = 1). Cure rates were similar across definitive antibiotic therapies (vancomycin-73.3%; TMP/SMX-88.9%; ceftaroline 50%; linezolid and doxycycline-100%). Three subjects died of MRSA disease; two definitively treated with vancomycin and one with ceftaroline. Conclusions: Vancomycin is the most commonly used agent in the treatment of invasive clindamycin-resistant CA-MRSA in children at our center. However, TMP/SMX and linezolid can be considered as oral options when completing treatment in select cases. Further work is needed to evaluate the optimal management of these infections.

## 1. Introduction

*Staphylococcus aureus* is a common pathogen responsible for a wide spectrum of disease including severe invasive infection. *S. aureus* was reported as the leading bacterial cause of death worldwide by the Global Burden of Disease Collaborative [1]. Notably, *S. aureus* displays distinct epidemiology in children relative to adults [1,2,3,4]. Specifically, adults with *S. aureus* bacteremia have higher rates of endovascular infection (12–20%) [5,6] relative to children (~3%) [7]. By contrast, children are disproportionately impacted by osteoarticular infection (69.7% of pediatric invasive *S. aureus*) and pneumonia [8]. Community-associated methicillin-resistant *Staphylococcus aureus* (CA-MRSA) disproportionately impacts the pediatric age group [9]. While vancomycin is recommended as first-line therapy in children by many experts, clindamycin is an effective agent for susceptible invasive CA-MRSA infections [10,11]. Clindamycin has the advantage of having excellent oral bioavailability and being well tolerated in children. However, clindamycin resistance in CA-MRSA has increased in many regions throughout the world [12,13,14,15,16]. Clindamycin resistance was reported to be as high as 38% among pediatric MRSA isolates in one large US center [17], complicating the management of this challenging infection. Moreover, rates of clindamycin-resistance have been reported to be higher in MRSA than MSSA infections in children in many centers including those in Europe, Israel and Australia [15,18,19]. Given shifts in the epidemiology of CA-MRSA in recent years [20,21,22], contemporary knowledge of the frequency and impact of resistance to clindamycin is of high clinical impact.

Vancomycin remains as first line therapy for serious MRSA infections in children [23]; however, given the risk of nephrotoxicity and need for close monitoring of renal function when using this drug, other agents may be preferable for definitive therapy. Moreover, the prolonged use of parenteral agents such as vancomycin can be associated with serious complications. Several studies report that central line associated bloodstream infections (CLABSI) associated with outpatient parenteral antimicrobial therapy (OPAT) for osteoarticular infections have occurred in up to 11–24% of patients [24,25,26,27]. Moreover, prolonged parenteral therapy in this diagnosis is associated with a higher readmission rate than use of oral agents [28], as well as non-infectious line-related complications (e.g., malfunction, displacement, etc.) in 23% [24]. Given the inability to use either β-lactams or lincosamides, there are limited options for oral therapy of serious clindamycin-resistant MRSA infections in children. Doxycycline has a high level of in vitro activity against most MRSA isolates and is commonly used for soft tissue infections; however, there are no prospective data regarding the use of this agent for serious invasive MRSA infections in children. Other oral agents such as trimethoprim-sulfamethoxazole (TMP-SMX) and linezolid have been suggested as possible step-down therapy for children with osteoarticular infections in areas with high clindamycin resistance [29,30]. However, specific data regarding the management and outcomes of invasive clindamycin-resistant MRSA infections in children treated with these agents are limited. We sought to describe the frequency, clinical features, management, and outcome of clindamycin-resistant CA-MRSA invasive infections at a large US children’s hospital.

## 2. Results

### 2.1. Overall S. aureus Disease Trends

**Figure 1:**
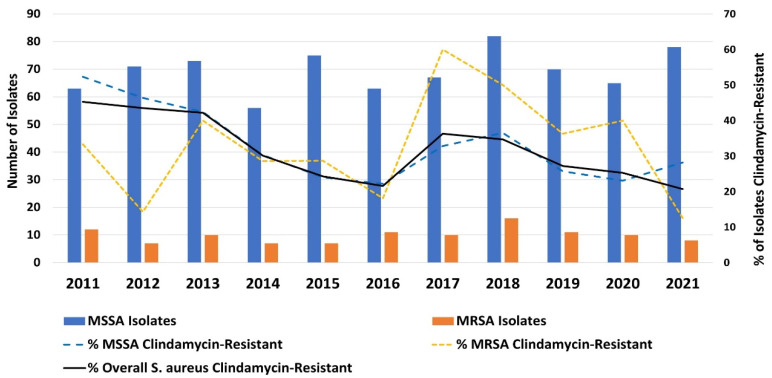
Invasive CA-*S. aureus*, 2011–2021.

A total of 872 invasive community-associated (CA)-*S. aureus* infections were identified from January 2011 to December 2021 through an active surveillance study of which 109 were CA-MRSA (12.5%, Figure 1); the annual proportion of MRSA varied during the study period without a clear trend. Overall, 35.7% of CA-MRSA were clindamycin-resistant; the proportion of MRSA which was clindamycin-resistant peaked in 2017 at 60%. An overall similar proportion of methicillin-susceptible *S. aureus* (MSSA) was clindamycin-resistant (32.1%). Clindamycin-resistant MRSA accounted for 3.9% of all invasive CA-*S. aureus* infections during the study period.

### 2.2. Use of Anti-MRSA Agents

**Figure 2:**
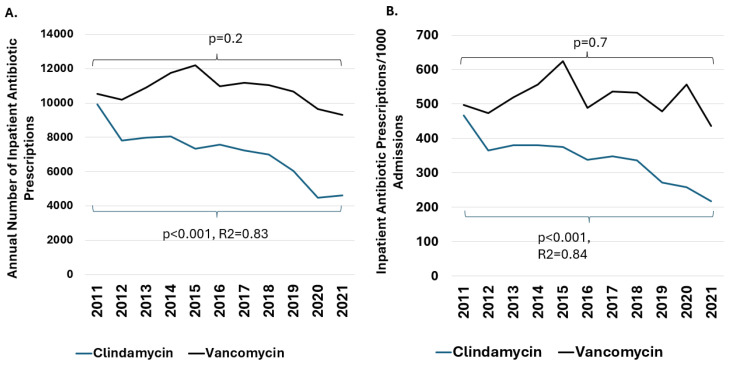
Trends in Inpatient Antibiotic Prescriptions. Panel (**A**). Annual number of inpatient prescriptions for vancomycin and clindamycin. Panel (**B**). Annual number of inpatient prescriptions for vancomycin and clindamycin adjusted for 1000 hospital admissions. A statistically significant decline in clindamycin prescriptions and prescriptions/1000 hospitalizations was observed (*p* < 0.001, R^2^ = 0.84). The frequency of vancomycin prescribing did not significantly change.

We sought to place our findings regarding the frequency of clindamycin-resistant MRSA in the context of prescribing anti-MRSA agents at our center. During the study period, there were 118,362 inpatient prescriptions for vancomycin and 78,024 for clindamycin across the hospital system. The annual number of inpatient vancomycin prescriptions was stable in the study period. By contrast, the frequency of clindamycin prescription declined significantly (*p* < 0.001, R^2^ = 0.84, Figure 2).

### 2.3. Clinical Features and Outcomes of Clindamycin-Resistant MRSA

Thirty-four children with clindamycin-resistant MRSA met full inclusion criteria and were studied in detail (Table 1). All subjects were admitted to our hospital. The most common diagnoses included osteomyelitis (n = 17, 50.0%) and deep abscesses (n = 7, 20.6%). Thirteen patients (38.2%) had positive blood cultures. All isolates were susceptible to vancomycin; three isolates were resistant to TMP-SMX (8.8%). Six subjects (17.6%) had documented receipt of clindamycin for a prior infection; one subject had a household contact with a history of clindamycin-resistant MRSA.

The most prescribed empiric antibiotic was vancomycin (n = 23, 67.6%). Statistically, 70.5% of subjects received empiric therapy with activity against the infecting isolate; 50% received clindamycin with or without other drugs as part of the empiric therapy regimen. Definitive antibiotics used for treatment included: vancomycin (n = 15, 44.1%), TMP-SMX (n = 9, 26.5%), linezolid (n = 7, 20.6%), ceftaroline (n = 2, 5.9%), and doxycycline (n = 1, 2.9%). There were no significant differences in age or surgical management of subjects treated with different regimens (Table 1). Patients treated definitively with vancomycin and ceftaroline were more likely to have positive blood cultures as well as longer durations of bacteremia and ICU admission than those receiving TMP-SMX or doxycycline. Overall, seventeen subjects (50%) were ultimately transitioned to oral antibiotics (Table 2). The median duration of intravenous (IV) therapy for all patients was 11.5 days (IQR 6–42 days) with a total therapy (IV + oral) time of 32 days (IQR 23–42).

Cure was achieved in 28 subjects (82.4%); cure rates were numerically comparable across definitive antibiotic therapies. Recurrence occurred in two patients treated with vancomycin (13.3%) and one patient treated with TMP-SMX (11.1%). Three deaths occurred of which two were treated with vancomycin and one with ceftaroline. One patient treated with vancomycin had a co-infection with influenza and only received one dose of vancomycin; this patient died on the day of admission. The second patient had disseminated CA-MRSA infection including pneumonia and mediastinitis and was treated with vancomycin but died 7 days later. The third deceased patient had necrotizing pneumonia, bilateral lower extremity osteomyelitis, and bacteremia. This patient was initially treated with vancomycin and clindamycin before being transitioned to ceftaroline and then succumbed to their disease 25 days later.

### 2.4. Infection in Non-Critically Ill Children

Subjects with illness not requiring intensive care unit (ICU) admission were examined in sub-analyses to adjust for the potential impact of severity of disease on antibiotic choice and outcomes (Table 3). Subjects receiving vancomycin for definitive therapy more often had positive blood cultures (*p* = 0.01) as well as longer durations of hospitalization (*p* = 0.08); diagnoses were similar across definitive therapy choices. There were no significant differences in cure rates among non-critically ill children receiving definitive therapy with vancomycin, TMP-SMX, linezolid or doxycycline.

### 2.5. Clindamycin-Resistant MRSA Osteomyelitis

As osteomyelitis represents the most common manifestation of invasive *S. aureus* disease in pediatrics, additional sub-analyses included 16 subjects with osteomyelitis without multisystem dissemination (Table 4). These patients received vancomycin most often for definitive therapy (n = 8, 50%), followed by TMP-SMX (n = 5, 31.3%) and linezolid (n = 3, 18.8%). Patients receiving vancomycin had longer durations of bacteremia as well as fever as inpatients. Overall, 14/16 subjects (87.5%) had surgical source control. Clinical cure rates across definitive treatment regimens were as follows: vancomycin, 87.5%; TMP-SMX, 80%; and linezolid, 100%.

## 3. Discussion

The emergence of clindamycin-resistant CA-MRSA is a major concern when treating invasive staphylococcal infections. The Committee on Infectious Diseases of the American Academy of Pediatrics recommends vancomycin for empiric treatment of suspected invasive *S. aureus* infections with the possible addition of an antistaphylococcal beta-lactam (e.g., nafcillin) for those who are critically ill. These recommendations state that empiric clindamycin should be reserved for non-life threatening infections without signs of sepsis and only used if clindamycin resistance rates are <15% in the community [23].

While our center has historically had high rates of MRSA [12], declines in CA-MRSA have been reported throughout the US in the past decade [8,21]. Of note, the frequency of clindamycin-resistance in both invasive MSSA and MRSA in our population was >30%, emphasizing the impact of these specific phenotypes. Importantly, the vast majority of children with clindamycin-resistant MRSA did not have prior receipt of clindamycin. Moreover, a high rate of clindamycin-resistance persisted despite declines in clindamycin prescriptions throughout our hospital system, highlighting the challenges in curbing antibiotic resistance as well as the community burden of this organism. It is conceivable that these data may reflect the existence of advantageous strains of clindamycin-resistant MRSA in our community. In a Korean study of MRSA bacteremia isolates obtained from children, 32.5% of isolates were clindamycin-resistant and isolates belonging to sequence type (ST) 5 and ST1 disproportionately contributed to clindamycin-resistance [31]. Of note, the majority of isolates in this study were healthcare associated, while the predominant genotype among CA-MRSA in the US is ST8 [32]. In a prior study of osteomyelitis including 361 children with *S. aureus* infection, demographic and laboratory variables at presentation were largely similar among those with clindamycin-resistant and -susceptible MRSA infection [33]. A notable exception, however, was that clindamycin-resistant MRSA osteomyelitis was associated with a higher rate of ICU admission. Such findings suggest that distinguishing clindamycin-resistant and -susceptible MRSA infections in clinical practice is challenging. Of note, a recent large study in Australia, a region with low rates of MRSA, reported temporal shifts in antimicrobial susceptibility in *S. aureus* bloodstream isolates [19]. While MRSA was relatively rare in the investigators’ population, the proportion of MRSA with clindamycin resistance steadily increased during the study period, emphasizing the wide ranging impact of this phenomenon.

While anti-staphylococcal β-lactams are the agents of choice for MSSA independent of clindamycin susceptibility, alternative agents are needed for clindamycin-resistant MRSA. While the number of subjects was relatively small, oral linezolid and TMP-SMX had similar outcomes in our series compared to vancomycin and avoids potential complications of prolonged parenteral therapy [24]. A single-center retrospective French study described the benefits of using linezolid as a first-line empirical therapy for osteoarticular infections (OAI) [34]. Additionally, Chen et al. [30] reported that 84.6% of patients with OAI due to gram-positive cocci (n = 13) responded favorably to linezolid as a step-down therapy. In our study, all patients treated with linezolid were cured, suggesting its efficacy as a step-down antibiotic therapy for select cases of clindamycin-resistant CA-MRSA osteomyelitis or other invasive infections. It should be acknowledged that, while it is well tolerated by most children, linezolid may be associated with adverse events including neutropenia, thrombocytopenia, lactic acidosis and peripheral neuropathy. Additionally, the cost of this agent can be prohibitive for certain populations. As such, providers must carefully weigh the risks and benefits, as well as affordability, of the available therapeutic approaches.

The recent guidelines from the Pediatric Infectious Diseases Society and the Infectious Diseases Society of America for the management of acute hematogenous osteomyelitis suggest vancomycin as the preferred antibiotic and daptomycin, ceftaroline or linezolid as alternatives for parenteral therapy of clindamycin-resistant MRSA infections [35]. Notably, these guidelines made no recommendation for the use of TMP-SMX given the very limited data in this clinical scenario. Similarly, the European Society for Pediatric Infectious Diseases bone and joint infection clinical practice guideline cautions regarding the limited published experience with TMP-SMX in these infections [36]. In their study of the efficacy of TMP-SMX in 38 cases of *S. aureus* OAI, Messina et al. [29] concluded that TMP-SMX is an appropriate step-down therapy. Importantly, in this prior study only three subjects were confirmed to have infections caused by clindamycin-resistant isolates. McDaniel et al. [37] observed no differences in treatment failure, hospital readmissions, or extension of therapeutic duration between patients with OAI treated with TMP-SMX vs. other antibiotics. While the McDaniel study was well-designed, this series only included four patients with MRSA. In our study, TMP-SMX step-down therapy was associated with a high cure rate (90% overall) in children with invasive MRSA after receiving IV therapy with other antibiotics for a median of 4.5 days in addition to having surgical drainage, highlighting the potential value of this approach in at least a subset of patients.

Some concerns when using TMP-SMX as a primary treatment include that it is known to cause several adverse drug events which may occur in up to 75% of patients, including cutaneous reactions, gastrointestinal intolerance, and cytopenias [38]. McDaniel et al. [37] reported that adverse drug events occurred in 41% of children with OAI treated with TMP-SMX for greater than two weeks duration. While our study was underpowered to assess adverse events associated with TMP-SMX, these data suggest that, in select pediatric patients with invasive MRSA infections, TMP-SMX is a reasonable therapeutic option. Moreover, the use of oral TMP-SMX avoids the potential hazards and costs of outpatient parenteral therapy [39] and may be particularly useful in resource limited settings. Importantly, patient tolerance and adherence to an oral therapy regimen, particularly among very young children requiring liquid formulations, must be considered when deciding route of therapy. It should be noted, however, that subjects in our series with longer durations of bacteremia were more often treated with intravenous agents (specifically vancomycin), perhaps reflecting provider discomfort with oral transition in the more severely ill.

Limitations to our work should be acknowledged. Foremost, our study is retrospective, introducing the potential for documentation bias, particularly with respect to reporting of prior clindamycin use as well as outcomes. Additionally, this is a single center study conducted in North America, impacting the generalizability of our findings to practices in other regions. The findings of this study may also have limited impact in centers with low rates of clindamycin use. The heterogeneity in infectious diagnoses makes interpretation of outcome data challenging. Our study population is relatively small decreasing its overall power to draw definitive conclusions and potentially introducing imprecision with respect to effect size; however, a strength of our work is that these data were derived from a large surveillance study with access to nearly comprehensive clinical metadata. Moreover, to our knowledge, this study represents the largest series of invasive clindamycin-resistant MRSA infections in children. 

## 4. Methods

### 4.1. Study Subjects

Cases of invasive clindamycin-resistant CA-MRSA infections in children cared for at a 973-bed tertiary US children’s hospital from January 2011 to December 2021 were prospectively identified through an ongoing *S. aureus* surveillance study which has been previously described [12]. Briefly, all *S. aureus* isolates identified by the hospital’s clinical microbiology laboratory in the routine course of care are sub-cultured, transported to the Infectious Diseases Research Laboratory and stored frozen in horse blood pending additional characterization. Clinical and demographic metadata associated with each isolate are prospectively recorded and stored securely in an electronic database. The Surveillance Study captures isolates identified in cultures obtained at three geographically distinct campuses in our hospital system. Isolates were routinely tested for susceptibility to oxacillin, clindamycin, trimethoprim-sulfamethoxazole and vancomycin by the hospital clinical microbiology laboratory using standard methods. Our center has historically had high rates of endemic CA-MRSA; in work from the mid-2000s at our institution, MRSA accounted for >60% of community-associated (CA)-*S. aureus* infections at its peak frequency [12,40,41]. For cases of clindamycin-resistant CA-MRSA, clinical and microbiologic data were obtained through review of the electronic medical record (EMR) and imported into an electronic case report form. Our center’s EMR includes all inpatient and outpatient encounters across an integrated system including three hospital campuses, twelve urgent care centers, and 51 affiliated primary care practices. Invasive infections included bacteremia, complicated pneumonia, septic arthritis, osteomyelitis, pyomyositis, and deep abscesses. For this study, only CA infections were eligible for inclusion [12]. CA infections were regarded as those occurring in (1) children lacking major medical comorbidities predisposing to frequent hospital admissions (e.g., congenital heart disease, malignancy, primary immunodeficiency, cystic fibrosis, etc.), and (2) with identification of *S. aureus* in the outpatient setting or within 48 h of hospital admission. Patients were excluded if they had an underlying comorbidity predisposing to frequent hospitalization; well-controlled asthma and eczema were not regarded as major comorbidities. All cases of clindamycin-resistant CA-MRSA were individually reviewed to confirm eligibility and cases with uncertainty regarding community-acquisition were discussed among investigators and a consensus reached.

### 4.2. Anti-MRSA Agent Use

Patients at our center with suspected CA-*S. aureus* infection (e.g., osteomyelitis) are typically treated empirically with an agent active against MRSA (i.e., vancomycin or clindamycin) [33]. The annual number of inpatient prescriptions for clindamycin and vancomycin in patients ≤ 18 years-old were obtained from the records of the hospital pharmacy. Temporal trends in prescribing and prescribing/1000 hospital admissions were examined using linear regression.

### 4.3. Outcomes and Analytic Concerns

Clindamycin-resistant CA-MRSA cases were stratified by definitive antibiotic treatment regimen and compared with respect to clinical features and outcomes. Definitive antibiotic treatment was regarded as effective antibiotic therapy, based on isolate susceptibility, that was used for >50% of the treatment course. Decisions regarding the specific choice of definitive antibiotic regimens as well as route of administration were at the discretion of the individual provider. Cure was defined as the resolution of symptoms, not requiring extension or change in antibiotics after completion of original antibiotic course, without needing additional surgical procedures or unscheduled visits related to initial infection. Duration of bacteremia and fever were reported in terms of calendar days. Categorical variables were compared with Fisher’s exact test and continuous variables with Wilcoxon Rank Sum or Kruskal Wallis tests as appropriate. Statistical analyses were conducted using STATA ver. 15 (STATACorp, College Station, TX, USA). Sub-analyses included a comparison of features and outcomes among the subset of children who were not admitted to the ICU to adjust for possible confounding related to critical illness. Given that osteomyelitis is the most common manifestation of invasive *S. aureus* disease in children [8], additional sub-analyses included an assessment of outcomes in children with clindamycin-resistant MRSA osteomyelitis stratified by definitive antibiotic choice. The local Institutional Review Board approved this study with a waiver of consent.

## 5. Conclusions

In conclusion, while vancomycin remains the preferred agent for the initial treatment of invasive clindamycin-resistant MRSA infections, TMP-SMX and linezolid may represent viable oral alternatives to complete treatment in highly select patients. While recognizing the limitations of our single-center retrospective design and sample size, our findings support the use of oral TMP-SMX or linezolid to avoid the complications and challenges of prolonged parenteral therapy in at least a subset of patients. Further multicenter work is needed to optimize the management of these infections.

## Figures and Tables

**Table 1:** Selected clinical features of children with invasive clindamycin-resistant MRSA infections and with definitive antibiotic treatment.

	All, n = 34	Vancomycin, n = 15	Ceftaroline, n = 2	TMP-SMX, n = 9	Linezolid, n = 7	Doxycycline, n = 1	*p*
Median Age, years (IQR) *	2.37 (0.79–8.7)	2.5 (0.86–8.3)	1.7 (0.03–3.6)	2.1 (1.7–11.2)	5.6 (0.3–12.9)	10.4	0.76
Gender (%) Male Female	16 (47.1) 18 (52.9)	6 (40) 9 (60)	1 (50) 1 (50)	5 (55.6) 4 (44.4)	3 (42.9) 4 (57.1)	1 (100) 0	0.89
Diagnosis (%) Osteomyelitis Septic Arthritis Bacteremia Pneumonia Pyomyositis Deep Abscess	17 (50) 1 (2.9) 1 (2.9) 6 (17.6) 2 (5.9) 7 (20.6)	8 (53.3) 0 1 (6.67) 3 (20) 1 (6.67) 2 (13.3)	1 (50) 0 0 0 1 (50) 0	5 (55.6) 0 0 1 (11.1) 0 3 (33.3)	3 (42.9) 1 (14.3) 0 2 (28.6) 0 1 (14.3)	0 0 0 0 0 1 (100)	0.57
ICU Admission	14 (41.2)	9 (60)	2 (100)	0	3 (42.8)	0	0.004
Median Duration of IV therapy, days (IQR)	11.5 (6–42)	42 (16–43)	30 (25–35)	4 (3–6) **	10 (9–12) **	6 **	<0.001 #
Median Duration of Oral Therapy, days (IQR)	21.5 (13–25)	n/a	n/a	17.5 (8–34)	22 (16–29)	16	0.9
Median Duration of Total Therapy (IV + Oral), days (IQR)	32 (23–42)	42 (28–52)	30 (25–35)	27 (12–42)	33 (28–42)	22	0.19
Positive Blood Cultures (%)	13 (38.2)	10 (66.7)	1 (50.0)	1 (11.1)	2 (28.6)	0	0.036
Median Duration of Bacteremia, days (IQR)	2 (1–3)	2 (2–3)	7	1	1 (1–1)	n/a	0.06
Surgical Source Control (%)	29 (85.3)	12 (80)	1 (50)	8 (88.9)	7 (100)	1 (100)	0.41
Cure (%)	28 (82.4)	11 (73.3)	1 (50)	8 (88.9)	7 (100)	1 (100)	0.35

* Continuous variables presented as median with interquartile range (IQR); categorical variables presented as n (%). ** Duration of IV therapy prior to oral transition; # When comparing duration of IV therapy in the vancomycin and ceftaroline groups, *p* = 0.42. IV—intravenous. n/a—not applicable

**Table 2:** Cases treated definitively with intravenous antibiotics compared with oral antibiotics.

	Intravenous n = 17	Oral n = 17	*p* Value
Median Age, years (IQR)	2.4 (0.9–6.3)	2.3 (0.8–11.2)	0.58
Positive Blood Culture (%)	10 (58.8)	3 (17.6)	0.03
Median Duration of Bacteremia, days (IQR)	2.5 (2–3)	1 (1–1)	0.01
Diagnosis (%) * Osteomyelitis Septic Joint Deep Abscess Bacteremia/Endovascular ** Pneumonia Pyomyositis	9 (52.9) 0 2 (11.8) 3 (17.6) 3 (17.6) 1 (5.88)	8 (47.1) 1 (5.9) 5 (29.4) 0 3 (17.6) 0	1 1 0.39 0.23 1 1
Cure (%)	12 (75)	16 (88.9)	0.38
Recurrence (%)	2 (11.8)	1 (5.9)	1
Deceased (%)	3 (18.8)	0	0.09

* Diagnoses are not mutually exclusive. ** Includes bacteremia without a source, septic thrombophlebitis (Lemierre’s Syndrome). No cases of endocarditis occurred.

**Table 3:** Comparisons of clinical features of non-critically ill children with invasive clindamycin-resistant MRSA infections stratified by definitive antibiotic treatment.

	Vancomycin, n = 6	TMP-SMX, n = 9	Linezolid, n = 4	Doxycycline, n = 1	*p*
Median Age, years (IQR)	8.5 (6.1–11.3)	2.1 (1.7–11.2)	9.3 (2.9–13.1)	10.44	0.5
Infectious Diagnosis					0.24
Osteomyelitis	5 (83.3)	5 (55.6)	2 (50)	0	
Septic Arthritis	0	0	1 (25)	0	
Bacteremia	1 (16.7)	0	0	0	
Pneumonia	0	1 (11.1)	0	0	
Deep Abscess	0	3 (33.3)	1 (25)	1 (100)	
Positive Blood Cultures (%)	5 (83.3)	1 (11.1)	1 (25)	0	0.01
Surgical Source Control (%)	4 (66.7)	8 (88.9)	4 (100)	1 (100)	0.49
Duration of Fever, days	6 (1–10)	1 (1–1)	2.5 (1–6)	1	0.27
Median Duration of Total Therapy (IV + Oral), days (IQR)	37.5 (28–52)	27 (12–42)	33.5 (32–38)	22	0.57
Length of Stay, days	14 (8–17)	5 (3–6)	9 (6.5–12)	6	0.08
Cure (%)	5 (83.3)	8 (88.9)	4 (100)	1 (100)	0.81

**Table 4:** Characteristics and treatment of clindamycin-resistant MRSA osteomyelitis.

	Vancomycin, n = 8	TMP-SMX, n = 5	Linezolid, n = 3	*p* Value
Age, years	5.7 (2.5–8.5)	11.2 (2.1–11.2)	7.5 (5.6–12.9)	0.6
ICU Admission	3 (37.5)	0	1 (33.3)	0.3
Positive Blood Culture	7 (87.5)	0	2 (66.7)	0.003
Duration of Bacteremia, days	2.5 (2–3)	n/a	1 (1–1)	0.007
Duration of Fever, days	9 (6–12.5)	1 (1–1)	3.5 (2–7)	0.02
Source Control	7 (87.5)	4 (80)	3 (100)	1
Number of Surgeries	1 (1–2)	1 (1–1)	2 (1–5)	0.14
Site of Osteomyelitis				0.49
Femur	3 (37.5)	1 (20)	1 (33.3)	
Tibia/Fibula	1 (12.5)	1 (20)	1 (33.3)	
Pelvis	2 (25)	0	0	
Other site	2 (25)	3 (60)	1 (33.3)	
Duration of IV Antibiotics, days	42 (35–46.5)	6 (3–8)	11 (10–13)	0.001
Duration of Total Antibiotics, days	47.5 (37.5–63)	42 (31–42)	42 (33–47)	0.46
Clinical Cure	7 (87.5)	4 (80)	3 (100)	1

## Data Availability

The original contributions presented in this study are included in the article. Further inquiries can be directed to the corresponding author.

## Abbreviations

CA—community-associated; MRSA—methicillin-resistant *Staphylococcus aureus*, TMP-SMX—trimethoprim-sulfamethoxazole.
